# Influencing factors of serum magnesium in CKD5 patients: A multicenter study in southern China

**DOI:** 10.3389/fpubh.2022.1047602

**Published:** 2022-12-14

**Authors:** Jiali Wang, Shujun Lin, Hong-Yan Li, Wenzhuang Tang, Yiping Liu, Tianbiao Zhou

**Affiliations:** ^1^Department of Nephrology, The Second Affiliated Hospital of Shantou University Medical College, Shantou, China; ^2^Department of Nephrology, Huadu District People's Hospital of Guangzhou, Southern Medical University, Guangzhou, China; ^3^Department of Blood Purification, The First Affiliated Hospital of Hainan Medical University, Haikou, China

**Keywords:** magnesium, chronic kidney disease, hemodialysis (HD), peritoneal dialysis (PD), multicenter study

## Abstract

**Introduction:**

Magnesium (Mg) disturbances are related to cardiac, bone, and renal patient mortality. In this study, we compared biochemical markers in hemodialysis (HD) and peritoneal dialysis (PD) patients and explored the influencing factors of serum Mg in stage 5 chronic kidney disease (CKD5) patients.

**Material and methods:**

All 598 patients with CKD5 from three medical centers in South China were recruited into this prospective cohort study from March 1, 2018, to January 31, 2021. Our study recorded the clinical characteristics and laboratory data of the patients.

**Results:**

Hemodialysis patients (0.99 ± 0.19 mmol/L) had a higher mean serum Mg level than PD patients (0.86 ± 0.20 mmol/L; *p* < 0.01). Regression analysis showed that only corrected calcium (Ca), phosphate (P), Ca/Mg, Ca × P, albumin (Alb), total protein and creatine (Cr) predicted Mg levels in CKD5 patients (*p* < 0.01). Ca/Mg predicts hypomagnesemia with 78% sensitivity and 85% specificity in CKD5 patients. The AUC value corresponding to Ca/Mg was 0.88.

**Conclusions:**

This multicenter study in southern China showed that for all CKD5 patients, corrected Ca and Alb had a significant positive effect on serum Mg, while Ca/Mg had a significant negative effect on serum Mg. In 123 HD patients, Ca × P was positively associated with Mg while Ca/Mg and P were negatively associated with Mg. In 398 PD patients, Ca × P, Alb, and total protein were positively associated with Mg while Ca/Mg and P were negatively associated with Mg. In 77 non-dialysis patients, corrected Ca, Cr, and total protein were positively associated with Mg while Ca/Mg was negatively associated with Mg. Furthermore, Ca/Mg might be another useful technique to monitor blood Mg levels in CKD5 patients.

**Clinical trial registration:**

ChiCTR1800014557.

## Introduction

Magnesium (Mg), the second most prevalent cation in cells after potassium, is involved in more than 300 enzymatic reactions and significantly impacts neurotransmitter release, oxidative stress prevention, bone metabolism, regulation of heart rhythm, and vascular tone (1). Mg ranges from 21 to 28 g in the human body. Around half of the total Mg in the human body is found in bones and teeth, with the remaining found in muscles or non-muscular soft tissue such as nerves and the brain (2). The intestines, bones, and kidneys of a healthy person maintain the homeostasis of Mg (3). Mg is obtained from the daily consumption of nuts, legumes, whole cereals, fruits, and so on (2). The excretion of serum Mg is significantly influenced by the kidney. In the kidney, 90%−95% of the filtered Mg is reabsorbed in the tubules, and 70%−80% of the ionized Mg is ultra-filterable (3).

In CKD patients, serum Mg abnormalities have been found (1). CKD patients may have hypomagnesemia or hypermagnesemia (1). Hypomagnesemia increases the risk of heart disease and a higher risk of hospitalization and death in dialysis patients (4, 5). Ventricular arrhythmias brought on by hypomagnesemia might be deadly (6). In addition, increased dietary Mg could reduce oxidative stress, proinflammatory response, and vascular calcification in the CKD animal model (7, 8). Furthermore, recent research has revealed that relatively high serum Mg concentrations may be beneficial for lowering cardiovascular risk, avoiding vascular calcification, managing hypertension, and controlling blood glucose in CKD patients (9, 10). Hypermagnesemia may reduce vascular calcification in dialysis patients. However, it can also cause pruritus and impair neuromuscular transmission, parathyroid gland function, and bone metabolism, resulting in bone mineralisation deficiency and renal osteodystrophy (11). Therefore, Mg is not an ion that should be neglected. It is necessary to have a deeper understanding of Mg abnormalities in CKD patients (10). Appropriate blood Mg monitoring is essential to keep its concentration within a reasonable range.

Patients with CKD may be able to compensate for the drop in Mg ultrafiltration brought on by the lower glomerular filtration rate by increasing their Mg urinary fractional excretion. However, fractional excretion cannot compensate for a substantial fall in estimated glomerular filtration rate (eGFR), particularly one below 30 ml/min. Thus, hypermagnesemia could be found in CKD patients. There are several reasons driving the development of hypomagnesemia in CKD. In CKD5 patients, dietary restriction is an important cause of hypomagnesemia. Limiting potassium consumption may decrease Mg intake as well, since potassium-rich foods are likewise high in Mg. Diabetes mellitus, proteinuria, and loop and thiazide diuretics all increase urinary Mg excretion. More significantly, due to reduced Mg reabsorption, tubular dysfunction or interstitial fibrosis may lead to urinary Mg loss (10). Serum Mg in dialysis patients has received increasing attention.

There are many reasons for abnormal blood Mg in CKD patients and exploring its influencing factors can help to understand the interrelationship between other laboratory indicators and serum Mg, clarify the clinical indicators that have a greater impact on serum Mg, help to speculate on the causes of serum Mg abnormalities, and finally provide a reference for correcting blood Mg abnormalities. However, only a few studies have been conducted in China (12–14). Reports of abnormal serum Mg levels and the factors affecting Mg in CKD patients have been inconsistent. Furthermore, there is no research on serum Mg levels in CKD5 patients in southern China. Clarifying the importance of Mg in CKD5 patients would improve outcomes and quality of life. Thus, we aimed to compare biochemical indicators in HD and PD patients and explore the factors influencing serum Mg in CKD5 patients.

## Materials and methods

### Study population

The protocol of this study was registered in the Chinese clinical trial registry (http://www.chictr.org.cn/showproj.aspx?proj=24882; No: ChiCTR1800014557). The inclusion criteria for participants were eGFR < 15 ml/min/1.73 m^2^ for at least 3 months with or without renal damage. Patients were excluded for the following reasons: eGFR ≥ 15 ml/min/1.73 m^2^, eGFR < 15 ml/min/1.73 m^2^ for < 3 months, and insufficient data. All 598 patients with CKD5 were recruited into this retrospective cohort study from the following three medical centers in South China from March 1, 2018, to January 31, 2021: the Second Affiliated Hospital of Shantou University Medical College, the Huadu District People's Hospital of Guangzhou of Southern Medical University, and the First Affiliated Hospital of Hainan Medical College. CKD5 was defined as an eGFR < 15 ml/min/1.73 m^2^. Demographic data, including sex, age, height, and weight, were recorded, and the body mass index (BMI) was calculated. The study was approved by the institutions of the Second Affiliated Hospital of Shantou University Medical College, the Huadu District People's Hospital of Guangzhou of Southern Medical University, and the First Affiliated Hospital of Hainan Medical College, and written informed consent was obtained from all the included participants.

### Parameters measurements

The clinical testing parameters were identified and extracted prospectively from three medical centers. The serum Mg level of three hospitals was detected by the xylidyl blue method; the normal reference range for Mg in this study was 0.70–1.1 mmol/L. Hypermagnesemia was defined as a serum Mg level of >1.1 mmol/L, and hypomagnesemia was defined as a serum Mg level of < 0.7 mmol/L. Phosphorus (P), calcium (Ca), immunoreactive parathyroid (PTH), potassium (K), sodium (Na), chlorine (Cl), creatinine (Cr), blood urea nitrogen (BUN), uric acid (UA), total protein, albumin (Alb), globulin, alanine aminotransferase (ALT), aspartic transaminase (AST), r-glutamyl transferase (r-GT), total bilirubin, direct bilirubin, indirect bilirubin, red blood cells (RBC), hemoglobin (Hb), mean corpuscular hemoglobin concentration (MCHC), white blood cells (WBC), platelet (PLT), serum iron, transferrin, ferritin, cholesterol (Ch), triglyceride (TC), high-density lipoprotein (HDL), low-density lipoprotein (LDL), C-reactive protein (CRP), high-sensitivity C reactive protein (hsCRP), creatine kinase (CK), creatine kinase isoenzyme MB (CK-MB), myoglobin, cardiac troponin T (CTnT), and alkaline phosphatase (ALP) were also determined. Corrected serum Ca was calculated as follows: measured serum Ca (mmol/L) + [40 – serum Alb (g/L)] × 0.02 (15, 16).

### Statistical analysis

In this study, SPSS 25.0 statistical software was used for statistical analysis. The measurement data was compared between HD and PD patients. The measurement information is presented in the form of mean ± SD. Stepwise multiple regression analysis was performed using blood Mg levels as the dependent variable and factors that were significantly correlated with serum Mg in the correlation analysis as independent variables. Ridge regression analysis was used if covariance between independent variables was found in the stepwise multiple regression analysis. The receiver operating characteristic (ROC) curve was used to analyse the indicators of Mg status. The significance level for all tests was *p* < 0.05.

## Results

### Baseline clinical data

A total of 598 stage 5 CKD patients were enrolled in this study. The sex distribution was 52.17% male and 47.83% female, and the mean age of the participants was 56.22 ± 14.11 years. The leading cause of CKD was glomerulonephritis (29.93%), followed by diabetic nephropathy (28.42%) and hypertensive nephropathy (14.38%). The CKD patient composition (HD/PD/non-dialysis) was 123/398/77. Most patients had normal serum Mg levels, whereas 13.47% had hypermagnesemia and 9.60% had hypomagnesemia. The baseline BMI was 22.55 ± 3.57. The serum Mg level was 0.89 ± 0.21 mmol/L. Other parameters are shown in Table 1.

**Table 1:** Baseline data of characteristics of 598 patients.

**Variables**	**Value**	**Variables**	**Value**
Age (year)	56.22 ± 14.11	Indirect bilirubin (μmol/L)	6.6 ± 3.47
BMI (kg/m^2^ )	22.55 ± 3.57	RBC (10^12^)	3.35 ± 0.95
Mg (mmol/L)	0.89 ± 0.21	Hb (g/L)	95.12 ± 24.66
iPTH (pg/ml)	90.23 ± 213.13	MCHC (g/L)	49.11 ± 74.19
Ca (mmol/L)	2.26 ± 0.27	WBC (10^9^)	9.25 ± 30.7
P (mmol/L)	1.81 ± 0.68	Plt (10^9^)	226.96 ± 91.46
K (mmol/L)	4.11 ± 0.86	Serum iron (μmol/L)	13.56 ± 37
Na (mmol/L)	138.82 ± 3.83	Transferrin (μmol/L)	9.08 ± 54.51
Cl (mmol/L)	98.72 ± 5.76	Ferritin (ng/ml)	286.63 ± 327.02
Cr (μmol/L)	905.43 ± 350.54	Ch (mmol/L)	4.82 ± 1.46
BUN (mmol/L)	54.02 ± 173.6	TG (mmol/L)	1.84 ± 1.6
UA (μmol/L)	439.5 ± 118.27	HDL (mmol/L)	1.22 ± 0.44
Globulin (g/L)	29.76 ± 5.76	LDL (mmol/L)	2.93 ± 1.01
Alb (g/L)	33.63 ± 6.06	CRP (mg/L)	24.07 ± 37.66
Total protein (g/L)	63.39 ± 8.03	hsCRP (mg/L)	19.31 ± 41.04
ALT (U/L)	21.61 ± 57.5	CK (U/L)	233.48 ± 372.06
AST (U/L)	24.9 ± 61.15	CK-MB (U/L)	15.61 ± 9.8
r-GT (U/L)	51.84 ± 98.53	Myo (ng/ml)	242.77 ± 190.11
Total bilirubin (μmol/L)	8.98 ± 5.77	CTnT (ng/ml)	1.68 ± 7.54
Direct bilirubin (μmol/L)	2.38 ± 3.63	ALP (U/L)	105.96 ± 105.03

BMI, body mass index; Mg, magnesium; iPTH, immunoreactive parathyroid; Ca, calcium; P, phosphorus; K, kalium; Na, natrium; Cl, chlorine; Cr, creatinine; BUN, blood urea nitrogen; UA, uric acid; Alb, albumin; ALT, alanine aminotransferase; AST, aspartic transaminase; r-GT, r-glutamyl transferase; RBC, red blood cell; HB, hemoglobin; MCHC, mean corpuscular hemoglobin contentration; WBC, white blood cells; PLT, platelet; Ch, cholesterol; TG, triglyceride; HDL, high density lipoprotein; LDL, low-density lipoprotein; CRP, C-reactive protein; hsCRP, hypersensitive C reactive protein; CK, creatine kinase; CK-MB, creatine kinase isoenzyme MB; Myo, myoglobin; CTnT, cardiac troponin T; ALP, alkaline phosphatase.

### Comparison of biochemical indexes between HD and PD patients

Compared to the mineral levels of HD and PD patients, the mean serum Mg levels of HD patients (0.99 ± 0.19 mmol/L) were higher than in PD patients (0.86 ± 0.20 mmol/L; *p* < 0.01). The mean PTH, corrected Ca, and K levels of HD patients were also higher than in PD patients (all *p* < 0.01). The mean Ca/Mg level of HD patients (2.28 ± 0.42) was lower than in PD patients (2.55 ± 0.49; *p* < 0.01). There were no significant differences in P and Ca × P levels between HD and PD patients.

Compared to the toxins and nutritional levels of HD and PD patients, the mean BUN, indirect bilirubin, total bilirubin, and MCHC levels of HD patients were higher than in PD patients (*p* < 0.01). The mean Cr, UA, RBC, Hb, Ch, and LDL levels of HD patients were lower than PD patients (*p* < 0.01). There were no significant differences between HD and PD patients in their levels of Alb, total protein, direct bilirubin, transferrin, TG, or HDL.

In comparison to the inflammatory biomarkers in HD and PD patients, HD patients had higher mean levels of ferritin, hsCRP, and globulin (*p* < 0.05; *p* < 0.01; *p* < 0.05). There were no significant differences in WBC and CRP levels between HD and PD patients.

There were no significant differences in age, BMI, ALT, AST, ALP, r-GT, CK, CK-MB, CTnT, and Myo levels between HD and PD patients (Table 2).

**Table 2:** Results of *t*-test analysis.

**Variables**	**Treatment**		***t***	***p*-Value**	**Variables**	**Treatment**		***t***	***p*-Value**
	**HD**	**PD**				**HD**	**PD**		
Age (year)	56.23 ± 13.59	56.01 ± 14.44	0.15	0.88	Indirect bilirubin (μmol/L)	6.91 ± 3.88	5.53 ± 2.72	2.98	0.00^**^
BMI (kg/m^2^)	20.98 ± 3.43	22.85 ± 3.57	−4.86	0.00^**^	RBC (10^12^)	3.17 ± 0.87	3.54 ± 0.92	−3.93	0.00^**^
Mg (mmol/L)	0.99 ± 0.19	0.86 ± 0.20	6.36	0.00^**^	Hb (g/L)	90.32 ± 24.90	99.73 ± 23.27	−3.71	0.00^**^
iPTH (pg/ml)	235.53 ± 439.67	57.24 ± 50.18	4.26	0.00^**^	MCHC (g/L)	125.83 ± 137.73	28.89 ± 7.82	7.8	0.00^**^
Ca (mmol/L)	2.35 ± 0.31	2.26 ± 0.23	2.92	0.00^**^	WBC (10^9^)	8.05 ± 4.12	9.87 ± 37.56	−0.54	0.59
Ca/Mg	2.28 ± 0.42	2.55 ± 0.49	−5.45	0.00^**^	Plt (10^9^)	218.64 ± 104.10	233.06 ± 89.54	−1.5	0.13
P (mmol/L)	1.81 ± 0.63	1.77 ± 0.67	0.53	0.6	Serum iron (μmol/L)	12.72 ± 9.55	13.71 ± 39.43	−0.17	0.87
K (mmol/L)	4.52 ± 0.91	3.93 ± 0.82	6.75	0.00^**^	Transferrin (μmol/L)	2.11 ± 4.19	13.73 ± 70.12	−1.07	0.29
Na (mmol/L)	137.70 ± 3.52	138.97 ± 3.58	−3.45	0.00^**^	Ferritin (ng/ml)	397.65 ± 358.30	268.82 ± 319.23	2.38	0.02^*^
Cl (mmol/L)	98.30 ± 4.60	97.60 ± 5.06	1.38	0.17	Ch (mmol/L)	4.49 ± 1.43	4.93 ± 1.37	−2.96	0.00^**^
Cr (μmol/L)	759.77 ± 299.17	966.06 ± 347.96	−5.93	0.00^**^	TG (mmol/L)	1.79 ± 1.85	1.87 ± 1.55	−0.48	0.63
BUN (mmol/L)	90.76 ± 228.54	22.53 ± 57.01	3.27	0.00^**^	HDL (mmol/L)	1.18 ± 0.45	1.24 ± 0.44	−1.31	0.19
UA (μmol/L)	384.26 ± 124.37	439.54 ± 98.81	−4.29	0.00^**^	LDL (mmol/L)	2.68 ± 0.99	2.97 ± 0.92	−2.84	0.00^**^
Globulin (g/L)	30.86 ± 6.23	29.57 ± 5.39	2.04	0.04^*^	CRP (mg/L)	24.91 ± 34.25	15.32 ± 26.17	0.88	0.38
Alb (g/L)	33.90 ± 5.47	33.66 ± 6.14	0.38	0.71	hsCRP (mg/L)	52.61 ± 60.87	8.93 ± 21.64	5.92	0.00^**^
Total protein (g/L)	64.76 ± 7.46	63.23 ± 7.89	1.88	0.06	CK (U/L)	171.85 ± 358.16	222.99 ± 276.93	−0.94	0.35
ALT (U/L)	34.57 ± 117.76	17.83 ± 23.56	1.54	0.13	CK-MB (U/L)	14.23 ± 8.73	16.88 ± 9.93	−1.8	0.07
AST (U/L)	35.82 ± 122.18	22.02 ± 29.78	1.22	0.22	Myo (ng/ml)	238.66 ± 140.62	359.46 ± 310.76	−1.77	0.09
r-GT (U/L)	67.46 ± 127.50	37.59 ± 63.71	1.06	0.29	CTnT (ng/ml)	0.89 ± 5.12	3.13 ± 11.75	−1.05	0.3
Total bilirubin (μmol/L)	9.96 ± 7.47	7.65 ± 3.85	2.86	0.00^**^	ALP (U/L)	117.64 ± 97.33	117.81 ± 127.09	−0.01	0.99
Direct bilirubin (μmol/L)	3.04 ± 5.13	2.12 ± 1.92	1.56	0.12					

BMI, body mass index; Mg, magnesium; iPTH, immunoreactive parathyroid; Ca, calcium; P, phosphorus; K, kalium; Na, natrium; Cl, chlorine; Cr, creatinine; BUN, blood urea nitrogen; UA, uric acid; Alb, albumin; ALT, alanine aminotransferase; AST, aspartic transaminase; r-GT, r-glutamyl transferase; RBC, red blood cell; HB, hemoglobin; MCHC, mean corpuscular hemoglobin contentration; WBC, white blood cells; PLT, platelet; Ch, cholesterol; TG, triglyceride; HDL, high density lipoprotein; LDL, low-density lipoprotein; CRP, C-reactive protein; hsCRP, hypersensitive C reactive protein; CK, creatine kinase; CK-MB, creatine kinase isoenzyme MB; Myo, myoglobin; CTnT, cardiac troponin T; ALP, alkaline phosphatase.

* *p* < 0.05.

** *p* < 0.01.

### Influencing factors of serum Mg in all CKD5 patients

The stepwise multiple regression analysis demonstrated that only corrected Ca, Alb, and Ca/Mg predicted serum Mg levels in CKD5 patients (*p* < 0.01). Corrected Ca, Ca/Mg, and Alb could explain 98.0% of the variation in Mg (*R*^2^ = 0.98). The model is valid (*F* = 520.078, *p* = 0.000 < 0.01) and equation of model is Mg (mmol/L) = 0.659 + 0.469^*^Corrected Ca (mmol/L) – 0.451^*^Ca/Mg + 0.008^*^Alb (g/L). In addition, there was no covariance problem (all VIF < 5) and no autocorrelation in the model (D-W = 2.12; Tables 3, **8**).

**Table 3:** Results of stepwise multiple regression analysis in 598 CKD5 patients.

**Variables**	**Unstandardized coefficients**		**Standardized coefficients**	***t***	***p*-Value**	**VIF**	***R* ^2^**	**Adjusted *R*^2^**	***F***
	***B***	**SE**	**β**						
(constant)	0.66	0.084	–	11.34	0.00^**^	–	0.98	0.98	*F*_(3, 32)_ = 520.08, *p* = 0.00
Ca	0.47	0.02	0.58	20.03	0.00^**^	1.32			
Ca/Mg	−0.45	0.01	−1.05	−35.94	0.00^**^	1.36			
Alb	0.01	0	0.23	8.86	0.00^**^	1.04			

Dependent variable: Mg.

D-W value: 2.12.

** *p* < 0.01.

### Influencing factors of serum Mg in HD patients with CKD5

Ridge regression analysis with a *k* value of 0.030 demonstrated a significant positive correlation between Ca × P and Mg. There was a significant negative correlation between Ca/Mg, P and Mg (Tables 4, **8**).

**Table 4:** Results of Ridge regression analysis in 123 HD patients.

**Variables**	**Unstandardized coefficients**		**Standardized coefficients**	***t***	***p*-Value**	***R* ^2^**	**Adjusted *R*^2^**	***F***
	***B***	**SE**	**β**					
(constant)	1.236	0.419	–	2.951	0.005^**^	0.802	0.721	*F*_(15, 37)_ = 9.979, *p* = 0.000
iPTH	0	0	−0.073	−0.869	0.39			
Ca/Mg	−0.274	0.045	−0.666	−6.035	0.000^**^			
P	−0.173	0.039	−0.716	−4.476	0.000^**^			
Ca × P	0.119	0.017	1.076	7.161	0.000^**^			
K	−0.01	0.016	−0.054	−0.633	0.53			
Cl	0.002	0.003	0.051	0.571	0.571			
Cr	0	0	0.001	0.011	0.991			
UA	0	0	−0.033	−0.335	0.739			
Total protein	0.003	0.002	0.144	1.386	0.174			
Indirect bilirubin	−0.005	0.004	−0.113	−1.349	0.186			
RBC	0.003	0.02	0.016	0.168	0.868			
Hb	0	0.001	−0.059	−0.623	0.537			
MCHC	−0.004	0.005	−0.07	−0.818	0.419			
Ch	0.015	0.009	0.145	1.76	0.087			
hsCRP	0	0	−0.071	−0.828	0.413			

Dependent variable: Mg.

** *p* < 0.01.

### Influencing factors of serum Mg in PD patients with CKD5

A positive correlation was found between Ca × P, Alb, total protein, and Mg using ridge regression analysis with a *k* value of 0.100. A significant negative correlation was found between Ca/Mg, P, and Mg (Tables 5, **8**).

**Table 5:** Results of Ridge regression analysis in 398 PD patients.

**Variables**	**Unstandardized coefficients**		**Standardized coefficients**	***t***	***p*-Value**	***R* ^2^**	**Adjusted *R*^2^**	***F***
	***B***	**SE**	**β**					
(constant)	0.844	0.112	–	7.527	0.000^**^	0.828	0.768	*F*_(12, 34)_ = 13.658, *p* = 0.000
iPTH	0	0	0.075	1.003	0.323			
Ca/Mg	−0.136	0.021	−0.507	−6.446	0.000^**^			
Ca × P	0.042	0.007	0.461	5.862	0.000^**^			
P	−0.038	0.017	−0.179	−2.281	0.029^*^			
K	0.012	0.018	0.054	0.695	0.492			
Cr	0	0	0.025	0.31	0.758			
UA	0	0	−0.136	−1.808	0.079			
Alb	0.005	0.001	0.207	3.314	0.002^**^			
Globulin	−0.001	0.002	−0.047	−0.758	0.454			
Total protein	0.002	0.001	0.112	3.387	0.002^**^			
Total bilirubin	0.002	0.004	0.042	0.575	0.569			
hsCRP	0	0	−0.051	−0.741	0.464			

Dependent variable: Mg.

* *p* < 0.05.

** *p* < 0.01.

### Influencing factors of serum Mg in non-dialysis patients with CKD5

The stepwise multiple regression analysis demonstrated that only corrected Ca, Ca/Mg, and Cr predicted serum Mg levels in non-dialysis patients with CKD5 (*p* < 0.01). The model is valid (*F* = 127.3732, *p* = 0.0000 < 0.01) and the equation of the model is Mg (mmol/L) = 0.3656 + 0.5019^*^Corrected Ca (mmol/L) – 0.3958^*^Ca/Mg + 0.0001^*^Cr (μmol/L) + 0.005^*^Total protein (g/L). In addition, there was no covariance problem (all VIF < 5) and no autocorrelation in the model (D-W = 2.0054; Tables 6, **8**).

**Table 6:** Results of stepwise multiple regression analysis in 77 non-dialysis patients.

**Variables**	**Unstandardized coefficients**		**Standardized coefficients**	***t***	***p*-Value**	**VIF**	***R* ^2^**	**Adjusted *R*^2^**	***F***
	***B***	**SE**	**β**						
(Constant)	0.3656	0.1179	–	3.1021	0.0029^**^	–	0.89	0.883	*F*_(4, 63)_ = 127.3732, *p* = 0.0000
Corrected Ca	0.5019	0.0377	0.6285	13.3298	0.0000^**^	1.2726			
Ca/Mg	−0.3958	0.0246	−0.8471	−16.0779	0.0000^**^	1.5893			
Cr	0.0001	0	0.1249	2.2613	0.0272^*^	1.7457			

Dependent variable: Mg.

D-W value: 2.0054.

* *p* < 0.05.

** *p* < 0.01.

### ROC analysis for hypomagnesemia in all CKD5 patients

**Figure 1:**
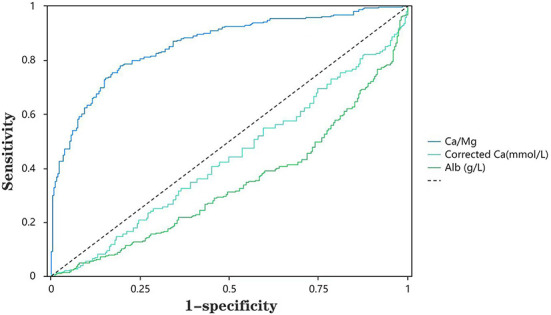
ROC curve of indicators for low magnesium.

The ROC curves were applied to evaluate the predictors of hypomagnesemia further. As shown in Table 7, we found that the sensitivity and specificity of Ca/Mg to predict hypomagnesemia were 78 and 85%, respectively (Table 7). The AUC value corresponding to Ca/Mg was 0.88, implying that Ca/Mg has a relatively high diagnostic value for hypomagnesemia. However, Ca and Alb are of less value for diagnosing hypomagnesemia. Figure 1 shows the ROC curves of Ca/Mg, Ca, and Alb for predicting hypomagnesemia in CKD5 patients, respectively (Figure 1).

**Table 7:** Results of ROC best bounds.

**Variables**	**AUC**	**Optimum boundary**	**Sensitivity**	**Specificity**	**Cut-off**
Ca/Mg	0.88	0.63	0.78	0.85	2.74
Corrected Ca	0.41	0.01	0.01	1	2.91
Alb	0.29	0	0	1	50

## Discussion

This multicentre study in southern China showed that in all CKD5 patients, corrected Ca and Alb had a significant positive effect on serum Mg; however, Ca/Mg had a significant negative effect on serum Mg. In HD patients, Ca × P was positively associated with Mg. However, Ca/Mg and P were negatively associated with Mg. In PD patients, Ca × P, Alb and total protein were positively correlated with Mg, while Ca/Mg and P were negatively correlated with Mg. In non-dialysis patients, Ca/Mg was inversely related to Mg, whereas corrected Ca, Cr, and total protein all had positive associations with Mg (Table 8). The ROC analysis for hypomagnesemia reported that Ca/Mg has a relatively high diagnostic value for hypomagnesemia. Similar studies have been conducted, but the findings of these studies are inconsistent. Cai et al. (12) found that serum Mg is positively correlated with dialysis duration, Alb, Hb, TG, K, Ca, and P but negatively correlated with Na in 253 PD patients in central China. Tsai et al. reported that serum Mg is positively correlated with P but negatively correlated with CRP and PTH in 180 PD patients in northern China (13). Ye et al. showed that Mg is negatively associated with hypertonic dialysate but positively associated with BMI, Alb, and P in 402 PD patients in southern China (14). A recent study reported that serum Mg concentrations were independently correlated with serum K concentrations in 60 PD patients (17).

**Table 8:** Summary of influencing factors of serum Mg in different subgroups in this study.

	**All CKD5 patients** **(*n* = 598)**	**HD patients** **(*n* = 123)**	**PD patients** **(*n* = 398)**	**Non-dialysis patients** **(*n* = 77)**
Positive correlation	Corrected Ca, Alb	Ca × P	Ca × P, Alb, total protein	Corrected Ca, Cr, total protein
Negative correlation	Ca/Mg	Ca/Mg, P	Ca/Mg, P	Ca/Mg

This study showed that corrected Ca is positively associated with Mg in non-dialysis patients with CKD5, which means that patients with hypomagnesemia may also have hypocalcemia. This result is consistent with a study in Zhejiang, China (12). Mg is involved in the ion channels transporting Ca into and out of cells. Unsurprisingly, Mg deficiency may perturb Ca homeostasis (18). There are several factors that lead to hypocalcemia in patients with hypomagnesemia. The first one is hypoparathyroidism. Low serum Mg level inhibits hypocalcemia-induced PTH release. Immunoreactive PTH levels are normal or low in most hypomagnesemia-hypocalcemic individuals (18–20). The mechanism for this could include disruption of the phosphoinositol system and decreased adenylate cyclase activity, as both are Mg-dependent (18). The second factor is resistance to PTH (20, 21). Mg deficiency could interfere with PTH-induced cyclic AMP production and lead to resistance to PTH, which may alter Ca homeostasis (22, 23). PTH-induced calcium release from bone is inhibited when plasma Mg falls below 1 mg/dl (20). The third factor is vitamin D deficiency. Impaired PTH secretion and a direct effect of Mg depletion on the kidney could impair vitamin D metabolism (23, 24). Moreover, low plasma vitamin D could lower Ca levels. In summary, hypoparathyroidism, PTH resistance, and vitamin D insufficiency could cause hypocalcemia in hypomagnesemia individuals.

In our study, Ca/Mg significantly negatively affected serum Mg and had a relatively high diagnostic value for hypomagnesemia in CKD5 patients. Serum Mg levels do not necessarily indicate total body Mg status (25). A study reported that the blood Mg/Ca ratio might be a more useful and sensitive measure of Mg than the serum Mg level alone (26). Our study found that Ca/Mg has a high diagnostic value for serum Mg levels. Therefore, the Ca/Mg may also be another practical method to assess blood Mg levels in CKD patients.

This study showed that P is negatively associated with Mg in PD patients, suggesting that elevating serum Mg concentrations may be able to reduce blood P in PD patients. Several studies have been conducted to evaluate the association between blood P levels and renal prognosis and mortality. According to a meta-analysis of 12 cohort studies including a total of 25,546 individuals, every 1 mg/dl rise in blood P level was related to renal failure (hazard ratio, 1.36) and death (hazard ratio, 1.20) (27). Another meta-analysis also reported that every 1 mg/dl rise in blood P level raised the chance of death by 18% (relative risk, 1.18; 95% CI, 1.12–1.25), highlighting the importance of P in CKD patients (28). Additionally, when serum P levels were already high in patients with stage 4–5 CKD, renal function deteriorated faster (29). Therefore, lowering blood P is important for the prognosis of CKD patients, and raising serum Mg could be a viable way to lower blood P.

Our study also found a positive correlation between blood Mg and the Ca × P product in both HD and PD patients. In non-dialysis patients, blood Mg was positively correlated with Cr, which indicates that hypermagnesemia may be associated with elevated Ca × P product and Cr. The effects of hypermagnesemia are still debatable. More cohort studies are needed to define the dangerous range of hypermagnesemia and its effect on complications and patient survival.

In this study, serum Mg levels were positively correlated with serum Alb in PD patients and positively correlated with total protein in both PD and non-dialysis patients, suggesting that serum Mg is related to nutritional status. Protein-energy wasting is common among dialysis patients and has emerged as a significant risk factor for morbidity and mortality (30). Alb and total protein are indicators of nutritional status. According to the “Gibbs-Donnan Effect,” Alb with an anionic charge during dialysis cannot pass through the semi-permeable membrane, producing an uneven charge and electric field, thus attracting positive ions, such as Mg ions, and preventing the Mg ions from moving across the semi-permeable membrane. Therefore, Alb decreases filterable Mg. In addition, serum Mg balance mainly depends on intestinal uptake and renal excretion, and patients with good nutritional status may have a high Mg intake. Therefore, the serum Mg level could reflect the Alb and total protein level to a certain extent.

The findings on the relationship between Mg and PTH have been conflicting in recent decades. In this study, no significant correlation was found in regression analysis between PTH and serum Mg, consistent with two retrospective studies, which enrolled 21,534 and 11,2017 HD patients (31, 32). However, some studies found that Mg had the opposite relationship with PTH (33).

This study was a multicenter study and included 598 CKD5 patients. The sample size of this study was relatively large. In this study, the mineral metabolism index, toxins and nutritional index, inflammatory index, and other indexes were included to explore the factors influencing serum Mg in CKD5 patients, and we also assessed the diagnostic value of several factors on serum Mg. Although this study was observational, this finding is important because few studies have investigated the importance of Mg. In clinical practice, serum Mg concentrations are not always routinely measured in all patients. This study highlights the importance of Mg and reminds us that proper monitoring of serum Mg concentrations is essential for CKD patients. In addition, this study found that Ca/Mg could be another practical way to assess blood Mg levels in CKD patients.

This study had several limitations. First, we did not investigate causality, as this was a cross-sectional and observational study. Second, we were unable to investigate the effect of Mg on all-cause mortality. Third, information on oral medications, such as Mg supplementation, was not collected. Therefore, the effect of oral medication on blood Mg concentrations was not explored in this study. Therefore, large-scale interventional studies are needed to clarify the importance of serum Mg and the effect of Mg supplementation in CKD patients.

## Conclusions

In conclusion, this multicenter study in southern China revealed a significant correlation between the serum Mg and corrected Ca, Ca/Mg, P, Ca × P, Cr, Alb, and total protein in CKD5 patients. This study also emphasized the importance of Mg and discovered that Ca/Mg might be another helpful technique to monitor blood Mg levels in CKD5 patients. More multicenter studies with large sample sizes will be required to guide therapy.

## Data availability statement

The original contributions presented in the study are included in the article/supplementary material, further inquiries can be directed to the corresponding author.

## Ethics statement

The study was approved by the institutions of the Second Affiliated Hospital of Shantou University Medical College, the Huadu District People's Hospital of Guangzhou of Southern Medical University, and the First Affiliated Hospital of Hainan Medical College, and written informed consent was obtained from all the included participants. The patients/participants provided their written informed consent to participate in this study. Written informed consent was obtained from the individual(s) for the publication of any potentially identifiable images or data included in this article.

## Author contributions

TZ contributed to the conception and design of the study and modified and polished the manuscript. JW, SL, H-YL, and WT were responsible for collection of data and performing the statistical analysis and manuscript preparation. YL and TZ were responsible for checking the data. JW wrote the manuscript. All authors were responsible for drafting the manuscript, read and approved the final version.
